# Assessment of Recent HIV-1 Infection by a Line Immunoassay for HIV-1/2 Confirmation

**DOI:** 10.1371/journal.pmed.0040343

**Published:** 2007-12-01

**Authors:** Jörg Schüpbach, Martin D Gebhardt, Zuzana Tomasik, Christoph Niederhauser, Sabine Yerly, Philippe Bürgisser, Lukas Matter, Meri Gorgievski, Rolf Dubs, Detlev Schultze, Ingrid Steffen, Corinne Andreutti, Gladys Martinetti, Bruno Güntert, Roger Staub, Synove Daneel, Pietro Vernazza

**Affiliations:** 1 University of Zurich, Swiss National Center for Retroviruses, Zurich, Switzerland; 2 Swiss Federal Office of Public Health, Berne, Switzerland; 3 Blood Transfusion Service, Swiss Red Cross Berne, Berne, Switzerland; 4 Hopital Cantonal Universitaire, Laboratoire Central de Virologie, Geneva, Switzerland; 5 Centre Hospitalier Universitaire Vaudois, Service d'Immunologie et d'Allergie, Lausanne, Switzerland; 6 Institut Dr. Viollier AG, Basel, Switzerland; 7 University of Berne, Institute of Infectious Diseases, Berne, Switzerland; 8 University Hospital, Department of Medicine, Zurich, Switzerland; 9 Institute for Clinical Microbiology and Immunology, St. Gallen, Switzerland; 10 University of Basel, Institute for Medical Microbiology, Basel, Switzerland; 11 Clinique de la Source, Lausanne, Switzerland; 12 Istituto Cantonale di Microbiologia, Bellinzona, Switzerland; 13 Labor Dr. Güntert, Lucerne, Switzerland; 14 Kantonsspital St. Gallen, Department of Medicine, St. Gallen, Switzerland; Centers for Disease Control and Prevention, United States of America

## Abstract

**Background:**

Knowledge of the number of recent HIV infections is important for epidemiologic surveillance. Over the past decade approaches have been developed to estimate this number by testing HIV-seropositive specimens with assays that discriminate the lower concentration and avidity of HIV antibodies in early infection. We have investigated whether this “recency” information can also be gained from an HIV confirmatory assay.

**Methods and Findings:**

The ability of a line immunoassay (INNO-LIA HIV I/II Score, Innogenetics) to distinguish recent from older HIV-1 infection was evaluated in comparison with the Calypte HIV-1 BED Incidence enzyme immunoassay (BED-EIA). Both tests were conducted prospectively in all HIV infections newly diagnosed in Switzerland from July 2005 to June 2006. Clinical and laboratory information indicative of recent or older infection was obtained from physicians at the time of HIV diagnosis and used as the reference standard. BED-EIA and various recency algorithms utilizing the antibody reaction to INNO-LIA's five HIV-1 antigen bands were evaluated by logistic regression analysis. A total of 765 HIV-1 infections, 748 (97.8%) with complete test results, were newly diagnosed during the study. A negative or indeterminate HIV antibody assay at diagnosis, symptoms of primary HIV infection, or a negative HIV test during the past 12 mo classified 195 infections (26.1%) as recent (≤ 12 mo). Symptoms of CDC stages B or C classified 161 infections as older (21.5%), and 392 patients with no symptoms remained unclassified. BED-EIA ruled 65% of the 195 recent infections as recent and 80% of the 161 older infections as older. Two INNO-LIA algorithms showed 50% and 40% sensitivity combined with 95% and 99% specificity, respectively. Estimation of recent infection in the entire study population, based on actual results of the three tests and adjusted for a test's sensitivity and specificity, yielded 37% for BED-EIA compared to 35% and 33% for the two INNO-LIA algorithms. Window-based estimation with BED-EIA yielded 41% (95% confidence interval 36%–46%).

**Conclusions:**

Recency information can be extracted from INNO-LIA-based confirmatory testing at no additional costs. This method should improve epidemiologic surveillance in countries that routinely use INNO-LIA for HIV confirmation.

## Abstract 2

Jörg Schüpbach and colleagues show that a second-generation Western blot antibody test used to confirm HIV infection can also be used to determine rates of recent HIV infection.

## Editors' Summary

**Background.:**

Since the first diagnosed cases of AIDS (acquired immunodeficiency syndrome) in 1981, the AIDS epidemic has spread rapidly. Now, 40 million people are infected with HIV (human immunodeficiency virus), the cause of AIDS. HIV infects and kills immune system cells, leaving infected individuals susceptible to other infectious diseases and tumors. The first, often undiagnosed, stage of HIV infection (primary HIV infection) lasts a few weeks and often involves a flu-like illness. During this stage, the immune system begins to respond to HIV by producing antibodies (proteins that recognize viral molecules called antigens). The time needed for these antibodies to appear on testing “seroconversion” (usually 6–12 weeks) is called the window period of the test; HIV antibody tests done during this period give false negative results. During the second, symptom-free stage of HIV infection, which can last many years, the virus gradually destroys the immune system so that by the third stage of infection unusual infections (for example, persistant yeast infections of the mouth) begin to occur. The fourth stage is characterized by multiple AIDS-indicator conditions such as severe bacterial, fungal, or viral infections, and cancers such as Kaposi sarcoma.

**Why Was This Study Done?:**

To monitor the AIDS/HIV epidemic and HIV prevention programs, it is necessary to know how many people in a population have been recently infected with HIV. Serologic testing algorithms for recent HIV seroconversion (STARHS) provide a way to get this information. Early during seroconversion, low levels of antibodies that bind only weakly to their viral antigens (low-affinity antibodies) are made. Later on, antibody concentrations and tightness of binding increase. STARHS calculate the number of recently infected people by analyzing data from special “detuned” HIV antibody assays (for example, a commercially available test called the BED-EIA) that preferentially detect low-concentration, low-avidity antibodies. This type of test cannot, however, be used to determine whether an individual has an HIV infection, because it will miss a substantial fraction of infected people. Diagnosing HIV in an individual person requires more sensitive tests for antibody detection. In this study, the researchers have investigated whether a test called INNO-LIA, which is already being used in some countries to diagnose HIV infection, can also provide information about the recency (newness) of HIV infections.

**What Did the Researchers Do and Find?:**

Between July 2005 and June 2006, 765 HIV infections were newly diagnosed in Switzerland. Using clinical and laboratory information collected at diagnosis, the researchers classified 195 of these infections as recent infections (occurring within the past year) and 161 as older infections. (The remaining infections could not be classified based on the available medical infomation.) The researchers then compared the ability of INNO-LIA (which measures antibodies to five HIV-1 antigens) and BED-EIA to distinguish recent from older HIV infections. BED-EIA correctly identified as recent 65% of the infections classified as recent based on the clinical information, and identified as older 80% of the infections classified as older based on the clinical information. In other words, this test was 65% sensitive (able to detect 65% of the truly recent infections as defined in this study) and was 80% specific (80% accurate in eliminating non-recent infections.) The two best algorithms (mathematical procedures) for converting INNO-LIA data into estimates of recent HV infections had sensitivities of 50% and 40% and specificities of 95% and 99%, respectively. Using actual test results and taking into account these sensitivities and specificities gave estimates of 35% and 33% for the proportion of the whole study population that had been recently infected. BED-EIA gave an estimate of 37%. Finally, a widely used window-based algorithm for recency estimation that uses the numbers of cases that are defined as recent by BED-EIA and the length of the window period for BED-EIA to calculate the annual number of new infections in populations indicated that 41% of the whole study population had been recently infected.

**What Do These Findings Mean?:**

These findings indicate that numbers of recent HIV infections can be extracted from the INNO-LIA HIV diagnostic test and are comparable to those obtained using a window-based algorithm. The test could, therefore, provide a cost-effective means to improve HIV surveillance in countries like Switzerland that already use it for HIV diagnosis. However, because this approach relies on knowing the sensitivity and specificity of the INNO-LIA algorithms, which may vary between populations, the use of these algorithms to estimate numbers of recent HIV infections must be preceded by an assessment of their sensitivity and specificity in each new setting.

**Additional Information.:**

Please access these Web sites via the online version of this summary at http://dx.doi.org/10.1371/journal.pmed.0040343.

- HIV InSite has comprehensive information on all aspects of HIV/AIDS, including fact sheets on the symptoms of HIV infection, HIV testing, and a chapter on laboratory tests for HIV antibodies
- NAM, a UK registered charity, provides information about all aspects of HIV and AIDS, including fact sheets on the stages of HIV infection and HIV testing
- The US Centers for Disease Control and Prevention (CDC) provides information on HIV/AIDS, including information on HIV testing and on HIV surveillance by the CDC (in English and Spanish)
- Information is available from Avert, an international AIDS charity, on the stages of HIV infection and on HIV testing
- Details on the US Centers for Disease Control and Prevention and the World Health Organiztion HIV classification systems are available from the US Department of Veterans Affairs

## Introduction

Assessment of the number of individuals with early HIV infection is needed for evaluation of the current HIV epidemic and preventive efforts targeted at the different transmission risk populations. Consequently, serologic testing algorithms for recent HIV seroconversion (STARHS) have been developed. These tests utilize the fact that both the concentration and affinity of HIV antibodies in early infection are lower than at later stages [1,2]. The fact that STARHS requires a special assay, which has a deliberately reduced sensitivity compared to HIV screening tests, restricts STARHS to epidemiologic studies. However, for systematic epidemiologic monitoring it would be advantageous if “recency” information (i.e. how recently the infection was acquired) could be simultaneously gained from the same tests being used to diagnose HIV infection.

Here we have investigated whether a second-generation Western blot assay (line immunoassay) provides recency information similar to that of a commercially available STARHS test, which is used for estimating the incidence of recent infections in a population and was originally developed by researchers at the US Centers for Disease Control and Prevention (CDC) [3]. The line immunoassay features standardized antigens of both HIV-1 and HIV-2 and three internal controls. It is currently being used increasingly as a replacement for the traditional first-generation HIV Western blot developed more than 20 years ago [4,5]. The test has recently become mandatory for confirmation of HIV infection and differentiation of HIV-1 and HIV-2 in Switzerland [6].

## Methods

### Patients and Specimens

It is estimated that several hundred thousand HIV screening tests are performed annually in Switzerland. Serum or plasma specimens from all individuals in whom HIV infection was newly diagnosed in Switzerland between 1 July 2005 and 30 June 2006 were selected for the study. By federal ordinance, an epidemiologic questionnaire on each newly diagnosed HIV infection has to be forwarded under code to the Swiss Federal Office of Public Health (SFOPH) by the patient's physician. Of relevance to the present study, the questionnaire contains items about clinical or laboratory signs of recent infection (e.g., symptoms of primary HIV infection, CDC staging [7], and seroconversion).

### Definition of Recent or Older Infection

For the purpose of this study an HIV infection was defined as recent on the basis of the physicians' information reported to the SFOPH and by the laboratory results obtained at the time of diagnosis of the HIV infection. Specifically, a recent infection was required to meet one or more of the following conditions. Definition 1: signs of primary HIV infection (PHI) at the time of diagnosis [8]; definition 2: a self-reported or documented negative result of a HIV screening test within 12 mo before HIV diagnosis; or definition 3, laboratory evidence of seroconversion at the time of diagnosis, i.e., a reactive fourth-generation HIV-1/2/O antibody/antigen combination screening test and a positive virus component test (HIV-1 p24 antigen or HIV-1 RNA) combined with a negative or indeterminate antibody assay (third-generation HIV-1/2/O antibody enzyme immunoassay, Western blot, or INNO-LIA). The time window during which an infection was defined as being recent thus was 12 mo. An infection was defined as older if symptoms of CDC stages B or C were present at the time of diagnosis and reported to the SFOPH [7]. Report of no symptoms (CDC stage A) or lack of staging information remained undefined and were either excluded from or included in the analysis depending on the respective evaluation as described under results.

### Tests for Recent HIV-1 Infection

All study samples were tested prospectively by the INNO-LIA HIV I/II Score assay (Innogenetics, Ghent, Belgium) in one of the 11 accredited Swiss HIV Confirmatory Labs or the Swiss National Center for Retroviruses (SNCR), the later being commissioned by the SFOPH to serve as the national HIV reference laboratory. INNO-LIA tests were conducted as a mandatory component of the SFOPH HIV testing system [6]. The INNO-LIA HIV I/II Score assay (INNO-LIA) is a Western blot–like line immunoassay that measures antibodies against recombinant proteins or synthetic peptides of HIV-1, HIV-1 group O, or HIV-2, which are coated as discrete lines on a nylon strip with plastic backing. As each test strip also contains three quantitative internal standards, a semiquantitative ranking of the different antibody reactions is possible [9,10].

All INNO-LIA assays were performed by trained laboratory personnel using the manufacturer's 16-h sample incubation protocol. Antibody reaction to each of the seven HIV antigen bands present on the INNO-LIA test strip (sgp120 [including group O peptides], gp41, p31, p24 and p17 of HIV-1, and sgp105 and gp36 of HIV-2) was assessed visually in nine laboratories and by the automated scanner–based LiRAS system (Innogenetics) in three laboratories. Based on the three internal standards, which define reaction levels of 0.5 (+/−), 1, and 3, the antibody reaction to each HIV antigen was classified into one of six possible intensity scores (0, 0.5 [for +/−], 1, 2, 3, or 4) according to the manufacturer's instructions.

Frozen aliquots (−20 °C) of all samples were furthermore sent under code to the SNCR and tested prospectively by the HIV-1 BED Incidence enzyme immunoassay (BED-EIA, Calypte Biomedical Corporation, Lake Oswego, Oregon, United States). BED-EIA was performed as a part of an ethically approved nationwide epidemiologic study (CH.A.T. survey) commissioned by the SFOPH. It aimed at elucidating the circumstances under which new HIV infections in Switzerland occurred (see http://www.bag.admin.ch/hiv_aids/00829/03471/index.html?lang=de for further information). All BED-EIA testing was performed in blinded fashion by a single, highly experienced person and using a Microsoft Excel-based program for automated result interpretation prepared for the study according to Calypte's instructions, which specify a cutoff of 0.8 normalized optical density units (OD_n_).

### Determination of INNO-LIA Window Periods

Window periods for INNO-LIA recency algorithms were determined based on results with 15 seroconversion panels with a total of 105 specimens (Boston Biomedica, West Bridgewater, Massachusetts, United States; panels PRB 903–904, 909–912, 916–919, 922–925, and 927). The INNO-LIA results on these panels were provided by Innogenetics. The raw window period was calculated as the difference in days between the first sample reactive in the most sensitive third-generation HIV-1/2 screening test (based on data from Boston Biomedica) and the first sample ruled “older” by the respective INNO-LIA recency algorithm. To these raw window periods was added a constant of 22 d, which corresponds to the estimated median seroconversion window of sensitive third-generation HIV-1/2 screening assays [11].

### Statistical Evaluation

**Figure 1:**
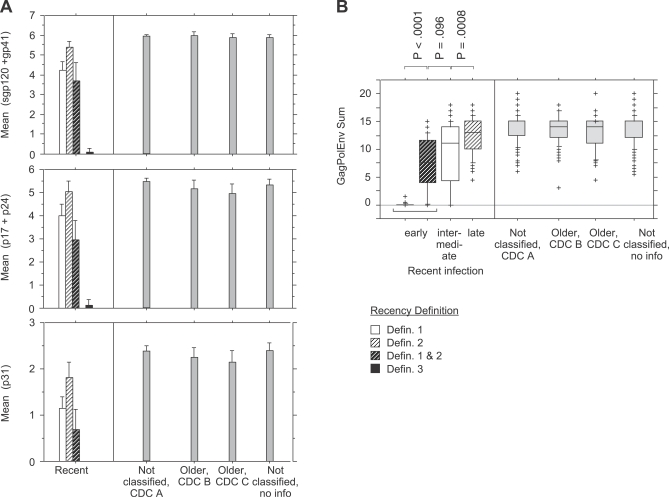
Antibody Reaction as Assessed by the INNO-LIA in Different Groups of Newly Diagnosed HIV-1 Patients (A) Sum of antibody scores to env antigens sgp120 and gp41 (top graph), gag antigens p24 and p17 (middle graph) and pol antigen p31 (bottom graph); bars represent means and error bars their 95% confidence intervals. Reaction is stratified according to recency definitions 1, 2, or 3 and other reported staging information (see Methods). (B) Correct order of recency definitions based on increasing antibody development. Box plots show sum of antibody scores to all five HIV-1 antigen bands. Boxes indicate the median and the 25th and 75th percentile. Horizontal lines located outside of, but closest to the boxes represent the 10th and 90th percentile, respectively. Outliers are plotted individually. The medians of the groups consisting of infections not classified coincide with the 75th percentile (upper limit of box). *p*-Values on top denote differences between subgroups of recent infections.

Differences in INNO-LIA reaction intensity in different study subgroups, as assessed in Figure 1, were compared by unpaired t-test. Specificity, sensitivity, diagnostic odds ratio, and logistic likelihood ratio of the BED-EIA and various tentative INNO-LIA HIV I/II Score algorithms for recency, as presented in Tables 1–3, were calculated by means of contingency tables and univariate and multivariate logistic regression analysis, as implemented by the StatView 5.0 program for Macintosh (SAS Institute, Cary, North Carolina, United States). Physicians' information was used as the reference standard for recency in all such evaluations. Estimates for the recent infection rates in Table 4 were obtained by the equation Recency Rate = (*n*_tested recent_/*n*_tested_ + %Specificity/100 − 1) / (%Sensitivity/100 + %Specificity/100 − 1). This equation is based on the relationship *n*_tested recent_ = *n*_true-recent_ + *n*_false-recent_, wherein *n*_true-recent_ = *n*_tested_ × Recency Rate × %Sensitivity/100 and *n*_false-recent_ = *n*_tested_ × (1 − Recency Rate) × (1 − %Specificity/100). Window-based estimates of the recent infection rate were performed by the equation (*n*_recent_/748) × (365/Window), shown with Table 5.

**Table 1:**
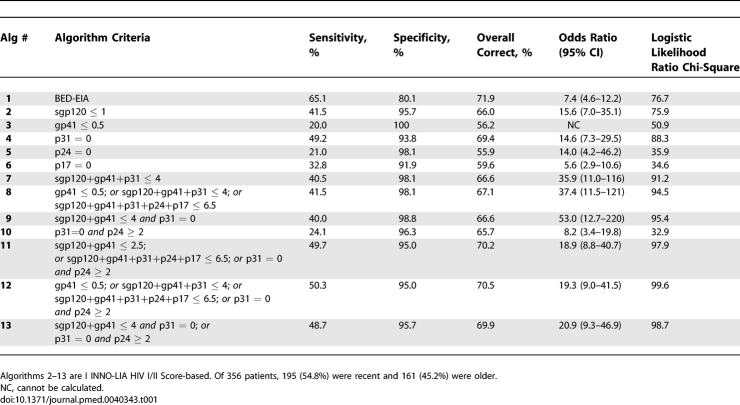
Performance of Various Algorithms for Detecting Recent Infection among Cases of Defined Recency Status

**Table 2:**
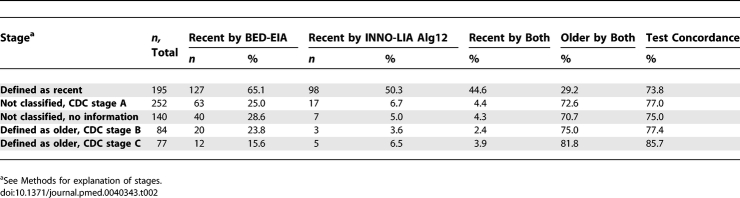
Diagnosis of Recent Infection and Test Concordance in Dependence of Stage

**Table 3:**
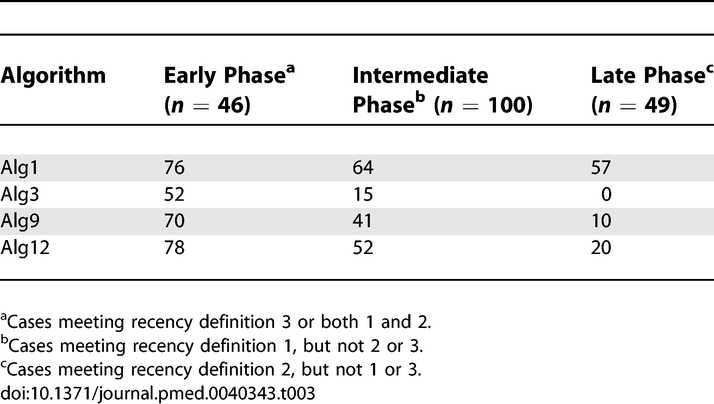
Percent Sensitivity of Various Recency Algorithms at Different Phases of Recent Infection

**Table 4:**
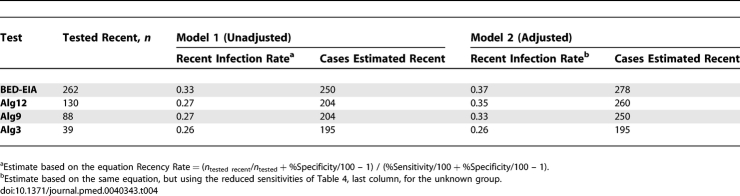
Estimation of the Overall Recent Infection Rate in the Study

**Table 5:**
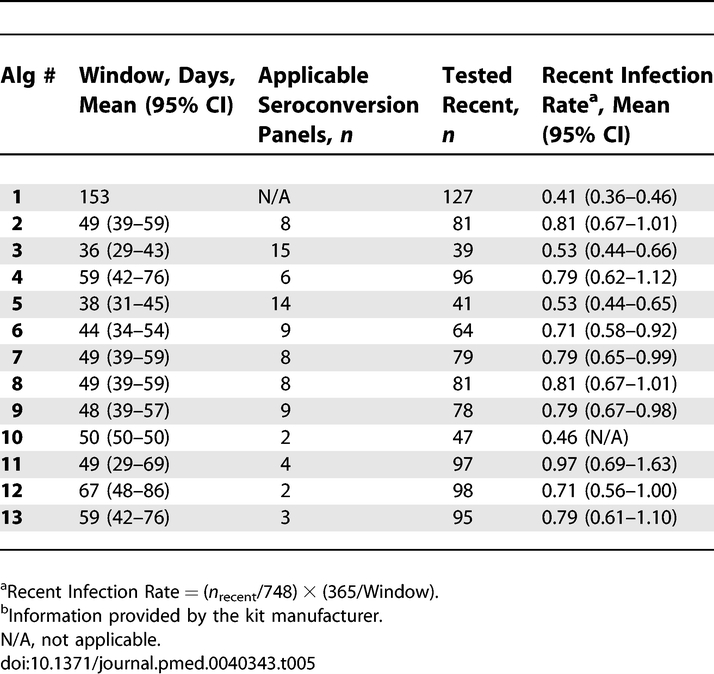
INNO-LIA Window Periods Derived from Seroconversion Panels and Estimates for the Recent Infection Rate

## Results

A total of 765 newly diagnosed HIV infections were reported to the SFOPH during the 12 mo of the study (July 05–June 06). Seventeen cases were excluded due to missing INNO-LIA results, thus leaving a total of 748 patients for evaluation (97.8%). All infections were by HIV type 1. A total of 195 patients met the definition of a recent infection as described in Methods, and 161 patients met the definition of an older infection. Of these, 84 were reported to be in CDC stage B and 77 in stage C. Remaining unclassified were 392 patients, 252 without symptoms (stage A) and 140 with no information provided. The main characteristics of the study population are summarized in Table 6.

**Table 6:**
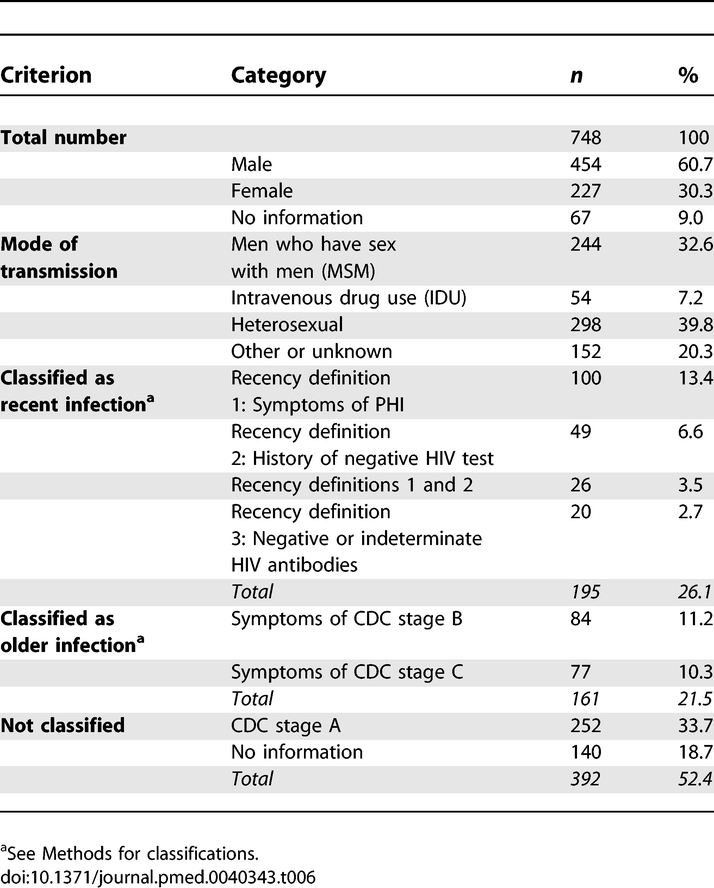
Characteristics of the Study Population

### INNO-LIA Reactivity in Dependence of Stage

INNO-LIA reactivity to env bands sgp120 + gp41, gag bands p17 + p24, and pol p31 in the different groups is summarized in Figure 1A. Infections defined as recent had lower mean antibody intensities to all antigens compared to all other groups (*p* < 0.001 in all comparisons). Those with both symptoms of PHI and a reported negative HIV test within the past 12 mo (thus meeting both recency definitions 1 and 2) had lower antibodies on average than those with reported PHI but no negative HIV test (recency definition 1), and the latter in turn had lower antibody levels than those with a history of a negative HIV test but no PHI symptoms (recency definition 2). Thus, those with both symptoms of PHI and a reported negative test represented an early phase, those with PHI symptoms alone an intermediate phase, and those with a documented negative test alone a late phase of recent infection (Figure 1B). Moreover, infections defined as recent based on their negative or indeterminate results in HIV antibody tests at the time of diagnosis (recency definition 3) showed the lowest antibody reactivity of all, thus classifying themselves as the most recent infections. Figure 1B also shows the *p*-values for the differences between the various subgroups of recent infection.

Antibody reactions in CDC stage A were similar to those in stages B or C except that antibodies to gag antigens were slightly higher than in stage C (*p* = 0.01), in agreement with established knowledge [12,13]. The 140 cases with no information were indistinguishable as a group from CDC stage A (Figure 1B) and like A exhibited a trend for higher antibodies to gag antigens compared to CDC stage C (for p17, *p* = 0.053).

### Prediction of Recent Infection in Patients with Defined Recency Status

A first evaluation of recency prediction by BED-EIA or INNO-LIA was based on the 356 cases classified either as recent or older as defined under methods. The BED-EIA classified 127 of the recent 195 infections as recent, exhibiting 65% sensitivity. The low sensitivity of the BED-EIA is due to the longer duration of the interval defined recent (12 mo in this study compared to 153 d in other studies using this test) [2,3,14]. The BED-EIA also classified 129 of the 161 older infections as older, exhibiting 80% specificity. Overall correct results amounted to 72%, the diagnostic odds ratio was 7.4, and the logistic likelihood ratio (LLR) Chi-square was 78.7 (Table 1).

Univariate logistic regression analysis showed that a low antibody score to any of the five HIV-1 antigen bands of the INNO-LIA strip was associated significantly with recent infection (*p* < 0.0001 for all, data not shown). Algorithms (Alg) 2–6 in Table 1 demonstrate the best power to discriminate between recent and older infection, as indicated by the highest LLR Chi-square achieved when varying the band intensity cut-off. Some of these algorithms exhibited similar or superior strength compared to BED-EIA, as shown by higher diagnostic odds ratios and LLRs. Alg2–6 were considerably more specific than was BED-EIA, but their sensitivities were lower, thus resulting in an overall lower percentage of correct results for INNO-LIA than for BED-EIA.

When using bands in combination and again optimizing cutoffs by means of logistic regression analysis, algorithms with around 98% specificity and 40% sensitivity were developed (Alg7–9). Based on the observation that seroconversion to p31 in Western blot occurs consistently later than to p24 [15,16], we also assessed this criterion alone (Alg10) or in combination (Alg11–13). Despite being a rather weak criterion on its own, Alg10 strongly increased sensitivity, albeit at some cost to specificity, when added to other criteria. Overall correct results by Alg11–13 amounted to around 70%, which was comparable to BED-EIA (72%). Multivariate logistic regression analysis adjusting for sex, transmission risk, and INNO-LIA reading mode (visual or automated) demonstrated no impact of these variables on the strength of the algorithms (unpublished data). Moreover, the reading mode had no significant impact on the INNO-LIA result. Although automated reading was associated with overall slightly lower intensity scores (*p* = 0.005, Mann-Whitney U test), probably due to the fact that the Liras software counts a score of 4 as 3, this reduction in mean scores had no impact on an algorithm's outcome, as an algorithm's cutoff is always placed at lower scores. For Alg12, for example, the odds ratio for automated versus visual reading for ruling an infection as recent was 1.11 (95% confidence interval [CI] 0.76–1.62; *p* = 0.60). There was also no difference in the case of single-band algorithms.

### Recency Rates in Different Patient Groups and Test Concordance

The proportion of patients ruled to have been infected recently by BED-EIA or INNO-LIA and the concordance of the two procedures in the different patient groups is shown in Table 2. Alg12 was selected for this comparison due to its overall highest rate of correct results (compare to Table 1). In all groups, BED-EIA found considerably more samples recent than did Alg12. Concordance between the two procedures was lowest among recent infections (74%) and highest in CDC stage C (86%). Note again that the relatively low sensitivity of the BED-EIA compared to other studies is due to the longer duration of the interval defined recent in this study.

We next investigated the sensitivity of selected algorithms of Table 1 for detection of the early, intermediate, or late phase of recent infection, as established in Figure 1B. The sensitivity of BED-EIA and all INNO-LIA algorithms decreased from early to intermediate to late phase (Table 3). In the early phase, INNO-LIA algorithms were similar in sensitivity to BED-EIA. In the intermediate and particularly the late phases of recent infection, INNO-LIA was considerably less sensitive than BED-EIA. Thus, the overall higher sensitivity of BED-EIA in Table 1 was due to its better recognition of the intermediate and late phases of recent HIV infection.

### Estimation of the Recent Infection Rate in the Entire Study Population

Given the more than 50% cases without reference recency information, the true proportion of recent infections (infections that occurred within the past 12 mo) in the study population was unknown. We estimated this value on the basis of the results of the different recency tests in the entire study population. The equation used for these estimates (see Methods) adjusts for a test's sensitivity and specificity, as determined in Tables 1 and 3.

The BED-EIA ruled 262 (35.3%) of the 748 infections as recent. When entering the sensitivity and specificity determined in Table 1 into the equation, a proportion of 33% recent infections corresponding to 250 cases was calculated (Table 4, model 1). Recency estimates by Alg12 and Alg9 were markedly lower, but yielded identical results to each other (27%, 204 cases), despite their considerable difference in the actual number of infections diagnosed as recent (130 versus 88). For Alg3 which according to Table 3 identifies only early recent infections and did not detect any further recent cases outside the group defined as recent, the estimate was remarkably close (26%).

Application of the sensitivity and specificity rates of the reference group of Table 1 to the unknown group is, however, feasible only if both groups are composed similarly; otherwise bias is introduced. Figure 1B shows that there is indeed considerable heterogeneity. Regarding their overall antibody reaction the unknown group differs significantly from the reference group of Table 1 (Mann-Whitney U test, *p* < 0.001). The unknown group has very little overlap with the early and intermediate phases of recent infection (Figure 1B). Thus, if the nonclassified infections include any recent infections at all, these are likely to be in the late phase of recent infection. It is therefore appropriate to use the reduced sensitivity rates shown in the last column of Table 3 for this group. Specificity, in contrast, was very similar among the different groups, in particular as regards INNO-LIA (cf. Table 2). Hence, by doing a mixed calculation, i.e., applying the sensitivity of Table 1 to the reference group and the reduced sensitivity of late recent infection of Table 3 to the unknown group while applying the specificity of Table 1 to both groups, we obtained more appropriate estimates for the recent infection rate of the entire study (Table 4, model 2). Compared with model 1 the adjusted model 2 showed an only 12% higher rate of recent infections for the BED-EIA, but as much as 20%–30% higher rates for Alg9 and Alg12, in agreement with the fact that the sensitivity of BED-EIA is reduced less in the late phase of recent infection than is the sensitivity of INNO-LIA (compare to Table 3). With recency rates of 0.35 and 0.33, respectively, Alg12 and Alg9 now showed good agreement with BED-EIA (0.37), while the estimate by Alg3, which does not detect cases in the late phase of recent infection, remained unchanged at 0.26. Alg3 clearly underestimated recent infection.

### Window Periods and Window-Based Estimation of the Recency Rate

In order to determine the window periods of the different INNO-LIA algorithms we analyzed INNO-LIA results of 15 seroconversion panels (Table 5; see also Methods). Alg3 (gp41) was associated with the shortest window period among the single band algorithms, followed by Alg5 (p24), Alg6 (p17), Alg2 (sgp120), and Alg4 (p31). The time span of many panels was shorter than the window period of many algorithms. The resulting window information is thus based on too few measurements and not reliable. Nevertheless, we tentatively used this information for an independent estimation of the recent infection rate in our study population. With this approach, BED-EIA yielded a rate of 0.41 compared to 0.37 obtained by model 2 of Table 5. Alg3 (gp41) and Alg5 (p24), whose window periods are the most reliable, both yielded a rate of 0.53. Other INNO-LIA algorithms supported less well by seroconversion panel results yielded rates of up to 0.98, thus confirming that the respective windows are indeed too short.

## Discussion

We demonstrate that a patient's antibody reaction in the INNO-LIA HIV I/II Score assay contains information which, similar to the Calypte BED-EIA, allows a distinction to be made between recent and older HIV-1 infection, thus providing a tool for estimating HIV-1 incidence in a population. Because antibody reactions to the five HIV-1 antigens measured by the INNO-LIA evolve over time after the infection (Figure 1), we evaluated various algorithms for their ability to distinguish between recent and older infection in a cohort of 748 unselected HIV-1 infected patients newly diagnosed during a period of 12 mo in Switzerland. The best INNO-LIA algorithms ruled 40%–50% of the 195 infections defined as being recent (up to 12 mo duration of infection) as recent, while ruling 95%–99% of the 161 infections defined as being older as older. In comparison, a dedicated commercial recency test, the BED-EIA, ruled 65% of the recent samples as recent and 80% of the older samples as older (Table 1). Concordance of the two tests was 74%–86% based on stage (Table 2). Both tests exhibited about 80% sensitivity for the early phase of recent infection, while showing reduced sensitivity for the intermediate and, particularly, late phases of recent infection (Table 3). Based on the sensitivity and specificity rates thus obtained from patients of known recency status we estimated the proportion of recent infections in the entire cohort of 748 patients. Utilizing an adjusted model we obtained recency rates of 0.33 and 0.35 for two INNO-LIA algorithms and of 0.37 for the BED-EIA (Table 4), which was similar to the window-based estimate of 0.41 for the BED-EIA (Table 5). There was higher discrepancy for the INNO-LIA algorithms, due to still-unreliable window period estimates.

All STARHS reported to date utilize window periods, i.e., the mean time from infection to the first positive result of a test. Based on the number of cases ruled recent in a tested population and the length of the window period the annual incidence can be calculated. The approach in our study is fundamentally different. Based on reference information obtained from the treating physicians we first defined two subsets of patients. One subset consisted of patients infected within 12 mo prior to HIV diagnosis (recent infection), and the other of patients who had older infections based on symptoms of CDC stage B or C. We used these two reference subsets to determine the sensitivity and specificity of various recency algorithms (Tables 1–3). Based on the now-known sensitivity and specificity and the number of cases ruled recent by a given algorithm the proportion of recent infections in the entire study population was calculated (Table 4). Thus, while the classical STARHS approach requires reliable window information for the test but no clinical information on the patients tested, our approach requires reliable clinical reference information for a subgroup of patients but no window information for the test.

During seroconversion, an HIV-infected person progresses from seronegativity to a pattern of full reactivity to all major HIV proteins [15]. Defining HIV seroconversion or recent infection as anything less than a full antibody reaction pattern may thus seem trivial. However, as disease progression is associated with decreasing concentrations of many antibodies, there is a second, late phase in HIV infection with incomplete patterns or submaximal concentrations of antibodies [12,13,17–20]. The detection of patients in recent infection by means of differential antibody reactivity thus requires a careful optimization of candidate algorithms with the aim to maximize correct assessment. The best INNO-LIA algorithms compared well with the BED-EIA regarding power to discriminate between recent and older infection and were considerably more specific than the BED-EIA (Table 1).

Unlike the BED-EIA, which due to its deliberately reduced sensitivity compared to HIV screening tests has no role in the diagnosis of HIV infection, the INNO-LIA is an excellent test for confirmation of HIV infection and is superior to (and less expensive than) Western blot for differentiating between HIV-1 and HIV-2 infection [9,10]. Early identification of HIV-2 infection is becoming increasingly important, as HIV-2 cannot be quantitated by any of the FDA-approved tests for HIV-1 RNA measurement [21] and is naturally resistant to several commonly used antiretroviral drugs [22–25]. The INNO-LIA HIV I/II Score assay is CE marked, i.e., approved for diagnostic use in the European Union, but currently cannot be used in the US, as the test is not FDA approved. INNO-LIA based STARHS is not a reasonable option for low-resource countries that use less expensive combinations of HIV screening tests for confirmation of HIV infection.

The recency information provided by the INNO-LIA comes at no additional costs and can be integrated easily into existing national HIV reporting systems. The number of cases for which recency information is available will thus be higher than in systems in which recency information must be gained from costly epidemiologic studies. This fact should result in HIV-1 incidence estimates of overall higher quality. The multicenter nature of the present study and the employment of both visual and automated strip evaluation in the 12 collaborating labs, with no apparent impact on the result, suggests that INNO-LIA-based recency assessment can be transferred to other labs without loss of quality. Studies to clarify this point and to determine whether automated reading is advantageous are currently underway.

In order to obtain an independent estimate of the recent infection rate in our study we also used a window-based approach (Table 5). The resulting infection rate of 0.41 of the BED-EIA was remarkably close to the 0.37 obtained by the adjusted model in Table 4. This agreement of results suggests that, by and large, the information from the physicians was reliable, although we cannot exclude the possibility of misclassification. For example, some PHI cases presenting with severe clinical manifestations may have been misclassified as older infections [26–29]. Altogether, the window based recency assessment by the BED-EIA however validates the sensitivity/specificity-based approach.

As the time span covered by many of the seroconversion panels was too short, sufficiently accurate window information is still lacking for most INNO-LIA algorithms. For Alg3 (gp41) and Alg5 (p24), window periods of 36 d and respectively 38 d were obtained, which is considerably shorter than the 153 d of the BED-EIA and consistent with the low sensitivity of INNO-LIA for the intermediate and late phases of recent infections (Table 3). Alg3 and Alg5 yielded an identical recent infection rate of 0.53, which is somewhat higher than that of BED-EIA (0.41). As the window periods are very short, a few days' difference can have a big effect when calculating an annual rate. For example, increasing the window periods of Alg3 or Alg5 by 6 d, as would result from using an average seroconversion interval of 28 d instead of 22 d (see Methods), would reduce the rate from 0.53 to 0.45. Testing of further panels covering extended time periods thus is urgently needed to define longer INNO-LIA windows with sufficient accuracy. Once established, window-based recency estimates will also be feasible for INNO-LIA algorithms and probably represent the preferred method. Different algorithms will also be usable in combination, which should result in more reliable estimates.

### Limitations

A possible current limitation of INNO-LIA-based recency testing may derive from the influence of infections by non-B subtypes of HIV-1. Like other European countries, Switzerland has a high rate of non-B infections [30] and the proportion of such viruses among newly diagnosed HIV-1 infections has now reached proportions of two-thirds in women and one-third in men (unpublished data from the Swiss genotypic resistance test database). Patients infected with such viruses may produce antibodies of reduced avidity to the subtype B antigens frequently employed in diagnostic tests, thus leading to low-reactivity patterns when tested and subsequent false diagnosis of recent infections. Such an effect has been observed with several early STARHS tests [31,32]. The BED-EIA, developed in order to overcome this problem, therefore features a branched peptide consisting of the immunodominant gp41 B-cell epitope of subtypes B, D, and E [3]. The fact that all INNO-LIA based algorithms were considerably more specific than the BED-EIA suggests that subtype interference should be less a problem for the INNO-LIA than it is for the BED-EIA. Systematic studies are planned to clarify this point.

Another known factor which may impair the specificity of STARHS consists of the reduced antibody concentrations associated with advanced disease stage (Figure 1). A further problem consists of cases that never reach a sufficiently high optical density to qualify as older infection [33]. The Joint United Nations Programme on HIV/AIDS Epidemiology Reference Group has recently issued a warning that the BED-EIA may overestimate HIV-1 incidence and should not be used for routine surveillance applications, and other investigations have also reported overestimation [14,34]. As the specificity of INNO-LIA based algorithms in patients with reported symptoms of AIDS is already high (Table 2), this concern should not be a problem for INNO-LIA. Nevertheless, studies addressing this question by means of well characterized patients of the Swiss HIV Cohort Study (SHCS) are planned.

As shown by Figure 1B, the nonclassified infections differ as group from the classified infections. Application of the sensitivity and specificity rates of the reference classified group to the unknown group will thus introduce bias. As a consequence, model 1 of Table 4, which does not correct for such bias, clearly underestimates the overall rate of recent infections. Although model 2 of Table 4 adjusts for the discrepancy, it is possible that the model still underestimates the true proportion of recent infections in the unknown group and, thus, the entire study population. Given the small difference between window-based and sensitivity/specificity-based recency rate estimates (0.41 compared to 0.37 for the BED-EIA), the influence of such possible further bias in the present study should be small, however. Nevertheless, as window-based recency assessment does not require knowledge of group composition, it should be the preferred method of INNO-LIA based recency assessment once INNO-LIA windows have been established with sufficient precision. Window-based recency assessment should also facilitate transfer of the method to other populations.

In conclusion, we provide proof of principle that a well-standardized line immunoassay for HIV confirmation and type differentiation also contains information on whether an HIV-1 infection is recent. The recency information provided by the INNO-LIA HIV I/II Score is available at no additional expenditure to countries in which this test is routinely used for confirmation of an HIV infection and HIV type differentiation. Utilization of this information could improve HIV surveillance in such countries. Because reliable window periods for INNO-LIA algorithms have not yet been established, INNO-LIA based recency assessment is currently restricted to the sensitivity/specificity approach described here. As the sensitivity and specificity of INNO-LIA algorithms may vary between different populations, it will be necessary to assess them anew when transferring the method to a different study population. Additional studies are needed to establish reliable window periods for the different INNO-LIA algorithms, to clarify the influence of HIV-1 subtype, and to further assess the specificity of the method in advanced disease.

## Supporting Information

Text S1 Flow Chart (26 KB PPT)

Text S2 STARD Checklist (258 KB PPT)

## Abbreviations

CI: confidence interval
EIA: enzyme immunoassay
LLR: logistic likelihood ratio
PHI: primary HIV infection
SFOPH: Swiss Federal Office of Public Health
STARHS: serologic testing algorithms for recent HIV seroconversion.
